# Two-in-One Biointerfaces—Antimicrobial and Bioactive Nanoporous Gallium Titanate Layers for Titanium Implants

**DOI:** 10.3390/nano7080229

**Published:** 2017-08-20

**Authors:** Seiji Yamaguchi, Shekhar Nath, Yoko Sugawara, Kamini Divakarla, Theerthankar Das, Jim Manos, Wojciech Chrzanowski, Tomiharu Matsushita, Tadashi Kokubo

**Affiliations:** 1 Department of Biomedical Sciences, College of Life and Health Sciences, Chubu University, Aichi Prefecture 487-8501, Japan; shekhar.nath@gmail.com (S.N.); cu33667@fsc.chubu.ac.jp (Y.S.); matsushi@isc.chubu.ac.jp (T.M.); kokubo@isc.chubu.ac.jp (T.K.); 2 Australian Institute for Nanoscale Science and Technology, Charles Perkins Centre, Faculty of Pharmacy, University of Sydney, Pharmacy and Bank Building A15, Sydney, NSW 2006, Australia; kdivakarla@gmail.com; 3 Department of Infectious Diseases and Immunology, Sydney Medical School, University of Sydney, Sydney, NSW 2050, Australia; das.ashishkumar@sydney.edu.au (T.D.); jim.manos@sydney.edu.au (J.M.)

**Keywords:** gallium ion, apatite formation, gallium titanate, Ti metal, simulated body fluid, antibacterial

## Abstract

The inhibitory effect of gallium (Ga) ions on bone resorption and their superior microbial activity are attractive and sought-after features for the vast majority of implantable devices, in particular for implants used for hard tissue. In our work, for the first time, Ga ions were successfully incorporated into the surface of titanium metal (Ti) by simple and cost-effective chemical and heat treatments. Ti samples were initially treated in NaOH solution to produce a nanostructured sodium hydrogen titanate layer approximately 1 μm thick. When the metal was subsequently soaked in a mixed solution of CaCl_2_ and GaCl_3_, its Na ions were replaced with Ca and Ga ions in a Ga/Ca ratio range of 0.09 to 2.33. 8.0% of the Ga ions were incorporated into the metal surface when the metal was soaked in a single solution of GaCl_3_ after the NaOH treatment. The metal was then heat-treated at 600 °C to form Ga-containing calcium titanate (Ga–CT) or gallium titanate (GT), anatase and rutile on its surface. The metal with Ga–CT formed bone-like apatite in a simulated body fluid (SBF) within 3 days, but released only 0.23 ppm of the Ga ions in a phosphate-buffered saline (PBS) over a period of 14 days. In contrast, Ti with GT did not form apatite in SBF, but released 2.96 ppm of Ga ions in PBS. Subsequent soaking in hot water at 80 °C dramatically enhanced apatite formation of the metal by increasing the release of Ga ions up to 3.75 ppm. The treated metal exhibited very high antibacterial activity against multidrug resistant *Acinetobacter baumannii* (MRAB12). Unlike other antimicrobial coating on titanium implants, Ga–CT and GT interfaces were shown to have a unique combination of antimicrobial and bioactive properties. Such dual activity is essential for the next generation of orthopaedic and dental implants. The goal of combining both functions without inducing cytotoxicity is a major advance and has far reaching translational perspectives. This unique dual-function biointerfaces will inhibit bone resorption and show antimicrobial activity through the release of Ga ions, while tight bonding to the bone will be achieved through the apatite formed on the surface.

## 1. Introduction

Titanium metal (Ti) and its alloys are widely used as orthopaedic and dental implants because of their high degree of mechanical strength and good biocompatibility. However, a polished Ti surface cannot bond to living bone by forming the requisite layer of thin fibrous tissue at the interface of living bone and the metal [1]. Roughened Ti surface is able to come directly into contact with living bone, but still does not bond to it adequately. In order to achieve stable fixation, various kinds of surface modifications introducing a bone-bonding capacity into Ti and its alloys have been attempted. A plasma spray coating of calcium phosphate is often used to induce the bone-bonding capacity. However, this method does not produce a uniform bioactive surface layer, because only the surface exposed to the plasma is coated, so the calcium phosphate is liable to decomposition in the living body over time. A coating of calcium phosphate has also been achieved by sputtering, sol-gel or alternative soaking [2,3]. However, once again the resulting coat is not stable in the body environment over time. The incorporation of calcium ions into the surface of Ti has also been attempted using ion implantation, micro-arc oxidation and hydrothermal treatment [4,5,6,7] to provide the capacity of apatite formation to the metal, while titania nanotubes have been formed by anodic oxidation to stimulate osteoblast cells [8]. However, these techniques require a special apparatus and are not suitable for devices of complex structure and/or of large size.

The solution and subsequent thermal treatment does not require any special apparatus and allows the formation of a uniform bioactive surface layer, even on the inner surface of a porous body [9,10]. It has been demonstrated that a bioactive surface layer composed of sodium titanate and rutile is produced on Ti by simply soaking it in NaOH solution at 60 °C for 24 h and then heating it at 600 °C for 1 h [11]. The treated metal spontaneously forms a bone-like apatite on its surface in the body environment and directly bonds to living bone through this layer [1,11,12,13,14]. These treatments were applied to the porous Ti layer of an artificial hip joint that was commercialized in Japan in 2007 [15]. The long-term survivorship of the NaOH- and heat-treated total hip arthroplasty (THA) was recently reviewed for 70 primary THAs, of whom 67 were available for follow-up periods of 8–12 years [16]. It was histologically observed that direct bonding to bone took place within 2 weeks and was maintained for at least 8 years. No implant exhibited any radiographic signs of evident loosening, and the overall survival rate was 98% at 10 years. 

However, for all the successful results of the NaOH- and heat-treated THAs, two joints were retrieved because of deep infection and periprosthetic femoral fracture [16]. In addition to bone bonding, the capacities of anti-bacterial and increasing bone density that may prevent bone infection and fracture are desirable for the next generation of THAs.

Recently, it was shown that various types of functional metal ions such as Sr, Mg and Ag can be incorporated into the Ti surface by modifying the NaOH and heat treatments [17,18]. In the case of Sr, the treated metal was expected to promote a growth of new bone surrounding Ti by releasing Sr ions and to form apatite on its surface by releasing Ca ions. Indeed, when the metal with Sr-containing calcium titanate was implanted into rabbit femur, it bonded to living bone in a shorter period of time compared to Ti with calcium titanate or sodium titanate, as expected [19,20]. Similar effects were observed for the Ti with Mg-containing calcium titanate that was produced by replacing SrCl_2_ with MgCl_2_ in the treatments [18,19,20]. While for Ag, Ti was soaked in a CaCl_2_ solution after the NaOH treatment, then subjected to a heat and AgNO_3_ treatment to form Ag-containing calcium titanate on its surface. This treated metal exhibited a strong anti-bacterial effect against Staphylococcus aureus as well as apatite formation; however, silver has long been recognised to be cytotoxic and recently recalled from many medical applications [21].

Gallium (Ga) is known for its inhibitory effect on calcium release from bone tissue, which is effective for preventing bone resorption. Gallium has also been shown to have clinical efficacy in suppressing osteolysis and bone pain and suggested as a treatment for osteoporosis [22]. In addition, recent studies have reported the anti-bacterial capability of Ga ions [23,24]. Valappil et al. reported the bactericidal activities of Ga-doped phosphate-based glass against both Gram-negative (*Escherichia coli* and *Pseudomonas aeruginosa*) and Gram-positive (*Staphylococcus aureus*, methicillin-resistant *Staphylococcus aureus*, and *Clostridium difficile*) bacteria [23]. Cochis et al. reported that the Ga-doped Ti produced by anodic spark deposition attained better bacterial inhibition against *Acinetobacter baumannii* than Ag-doped Ti by the same method [24]. In contrast with Ag, Ga can active metabolically by substituting Fe in many biological systems, due to the chemical similarities of Ga^3+^ with Fe^3+^ in terms of charge, ionic radius, and electronic configuration [25]. As a result, Ga exhibits these beneficial effects without inducing cytotoxicity [26,27,28,29].

We have designed novel Ga-containing nanostructured interfaces that are capable of sustainably realising gallium ions. In this way, we will achieve highly desired antimicrobial activity without compromising the ability of the implant to bind to bone. In fact, gallium ions are likely to further improve bone bonding ability and ultimately lead to improved bone quality. This improved effectiveness in stimulation of the bone formation is of particular value to achieve stable integration in osteoporotic of Dorr bone type environment. For these patients, conventional approaches reach a very high level of failure, which continues to increase with aging society, and for which traditional surfaces are suboptimal. Statistical data suggest that implant applications will skyrocket over the next few decades. The high number of surgeries that need to be repeated as implants fail to integrate in the patient’s body, becoming infected or ineffective (up to 17.5% of devices), imposes an additional and growing burden. Advances, such as that which we have developed, promoting rapid implant integration to improve function and reduce the risk of infection are therefore of great significance. The developed multifunctional interfaces will deliver rapid osseointegration of orthopaedic implants, and enable enhancement of the design of effective biomaterials in general.

## 2. Results

### 2.1. Surface Structures

**Figure 1:**
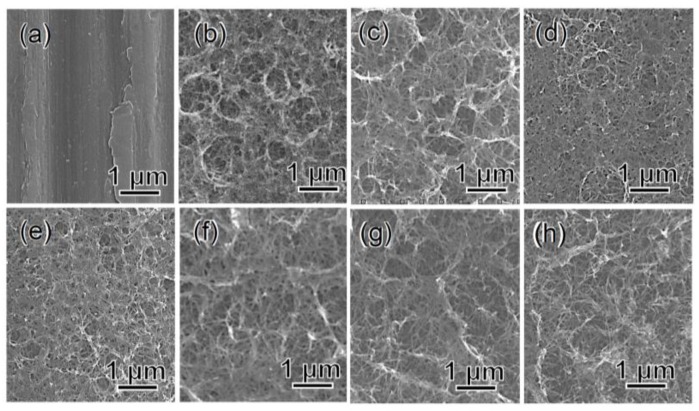
FE-SEM photographs of surfaces of Ti (**a**) untreated or subjected to (**b**) NaOH treatment, and subsequent (**c**) 100Ca + 0.05Ga and (**d**) heat treatment, and finally (**e**) water treatment, or (**f**) 100Ga after the NaOH treatment, and subsequent (**g**) heat and finally (**h**) water treatment.

The surface and cross-sectional FE-SEM (field emission scanning electron microscopy) observations showed that a fine network structure approximately 1 μm thick uniformly formed on the Ti surface with the first NaOH treatment, as reported in our previous work [30]. The nano-sized network morphology was retained even after the subsequent chemical and heat treatments, as shown in Figure 1a–h.

Table 1 shows the chemical composition of the Ti surfaces taken by EDX (energy dispersive X-ray spectrometer) analysis after each chemical and heat treatment. It should be noted that the results show averaged values of the graded compositions from the surface to the Ti substrate (later shown in Figure 5). The first NaOH treatment incorporated approximately 5.5% of the Na into the surface of the metal. When the metal was subsequently soaked in a mixed solution of 100 mM CaCl_2_ and 0.01 mM GaCl_3_, the Na was replaced with 3.5% Ca and 0.3% Ga such that the Ga/Ca ratio was 0.09. The ratio of Ga/Ca in the surface region increased with an increasing concentration of GaCl_3_ in the mixed solution. 8.0% of the Ga was incorporated into the surface layer when the metal sample was soaked in 100 mM GaCl_3_ solution after the NaOH treatment. 

The metal sample with 2.3% Ca and 0.6% Ga (Ga/Ca ratio 0.26) did not exhibit any change in Ca or Ga by subsequent heat treatment, but exhibited an appreciable decrease in Ca by subsequent water treatment. The metal sample with 8.0% Ga displayed little change in Ga by subsequent heat treatment, but a slight decrease in Ga resulted from the final water treatment. 

**Figure 2:**
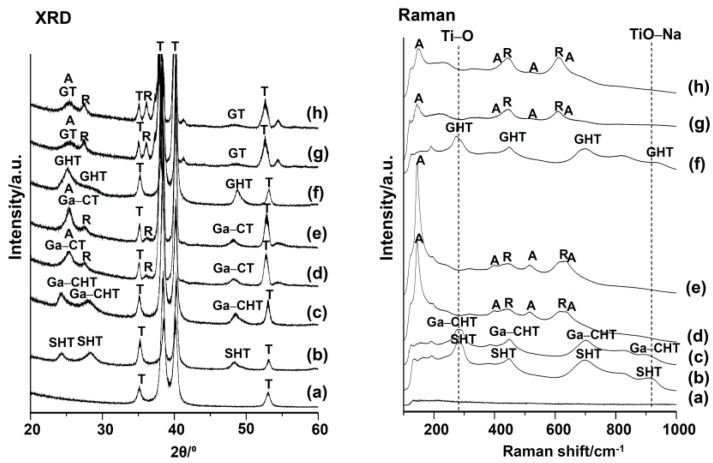
XRD and Raman spectra of surfaces of Ti (**a**) untreated or subjected to (**b**) NaOH treatment, and subsequent (**c**) 100Ca + 0.05Ga and (**d**) heat treatment, and finally (**e**) water treatment, or (**f**) 100Ga after the NaOH treatment, and subsequent (**g**) heat and finally (**h**) water treatment. T: α-Ti A: Anatase R: Rutile SHT: Na*_x_*H_2−*x*_Ti_3_O_7_ Ga–CHT: Ga*_x_*Ca*_y_*H_2-(3*x*+2*y*)_Ti_3_O_7_ GHT: Ga*_x_*H_2−3*x*_Ti_3_O_7_. Ga–CT: Ga*_x_*Ca_1−1.5*x*_Ti_4_O_9_, Ga*_x_*Ca_1−1.5*x*_Ti_2_O_4_, Ga*_x_*Ca_1−1.5*x*_Ti_4_O_5_ GT: Ga_2_TiO_5_.

Figure 2 shows the TF-XRD (thin-film X-ray diffraction) and FT-Raman (Fourier transform confocal laser Raman spectrometry) profiles of the sample surfaces subjected to NaOH, 100Ca + 0.05Ga or 100Ga, heat and final water treatments. The initial NaOH treatment resulted in broad XRD and Raman peaks attributed to sodium hydrogen titanate [31,32], Na*_x_*H_2−*x*_Ti_3_O_7_ (SHT). When the Ti was subsequently subjected to 100Ca + 0.05Ga treatment, the peak positions of SHT were essentially unchanged except for a slight shift in the Raman peak from approximately 920 to 900 cm^−1^. This indicates that the sodium hydrogen titanate was isomorphously transformed into gallium-containing calcium hydrogen titanate, Ga*_x_*Ca*_y_*H_2−(3*x*+2*y*)_Ti_3_O_7_ (Ga–CHT), by substituting for the Na ions with Ca and Ga ions. The Raman peak around 920 cm^−1^ was previously reported as Ti–O bonds involving nonbridging oxygen coordinated with Na ions [33], with its shift to a lower wave number potentially resulting from an exchange of Na ions with Ca and Ga ions. When the Ga–CHT was heat-treated, broad peaks at around 25° and 48°, as well as a peak of rutile around 27.5° in 2θ, appeared in the TF-XRD pattern. The broad peaks were well matched with those of the calcium titanate (CT), such as CaTi_2_O_4_ (JCPDS file 00-026-0333), CaTi_2_O_5_ (JCPDS file 01-072-1134), and CaTi_4_O_9_ (JCPDS file 00-025-1450), which were formed by the heat treatment of CHT [34,35,36]. This shows that Ga–CHT was transformed into gallium-containing calcium titanate (Ga–CT), such as Ga*_x_*Ca_1−*x*_Ti_2_O_4_, Ga*_x_*Ca_1−*x*_Ti_2_O_5_ and Ga*_x_*Ca_1−*x*_Ti_4_O_9_. Certain amounts of anatase were also formed by the heat treatment, as seen in the peaks in Raman spectra at around 150 and 520 cm^−1^. These crystalline phases were unchanged by the final water treatment. In contrast, gallium hydrogen titanate, Ga*_x_*H_2−3*x*_Ti_3_O_7_ (GHT), was formed on Ti surface when the metal was subjected to the 100Ga treatment after the NaOH treatment. The Raman spectrum in Figure 2f shows a slight shift of the peak at 290 cm^−1^ toward a lower wave number, implying that a portion of the Ti^4+^ in SHT was replaced by Ga ions. The GHT was transformed into gallium titanate, such as Ga_2_TiO_5_ (GT) (JCPDS file 00-020-0447), along with rutile accompanied by a small amount of anatase, which remained even after the final water treatment, as shown in Figure 2g,h.

**Figure 3:**
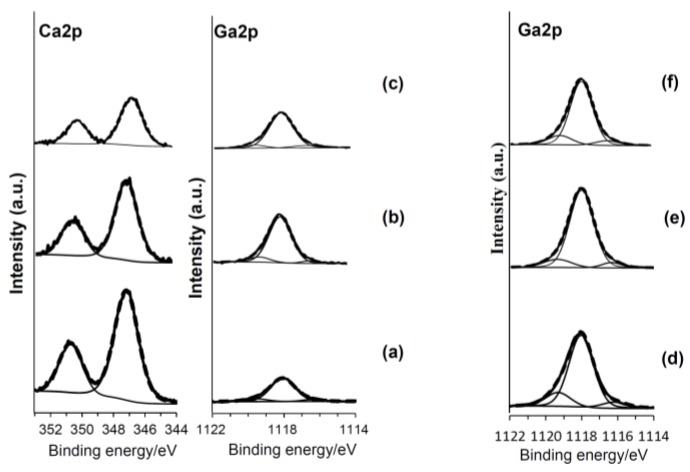
XPS profiles of Ca2p and Ga2p on Ti subjected to (**a**) 100Ca + 0.05Ga; (**b**) subsequent heat, and (**c**) final water treatment, or (**d**) 100Ga; (**e**) subsequent heat, and (**f**) final water treatment. Thick line represents spectrum. Thin lines represent deconvolution lines. Dot line represents composite line.

**Figure 4:**
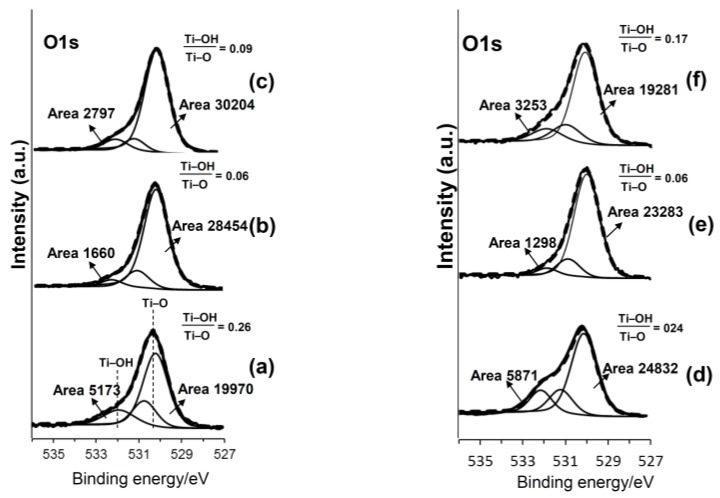
XPS profiles of O1s on Ti that was initially soaked in NaOH solution and then subjected to (**a**) 100Ca + 0.05Ga; (**b**) subsequent heat, and (**c**) final water treatment, or (**d**) 100Ga; (**e**) subsequent heat, and (**f**) final water treatment. Thick line represents spectrum. Thin lines represent deconvolution lines. Dot line represents composite line.

Figure 3 shows the high-resolution XPS (X-ray photoelectron spectroscopy) spectra of the Ca2p and Ga2p on the samples that were subjected to the NaOH and 100Ca + 0.05Ga or 100Ga treatments, subsequent heat treatment and final water treatment. The metal subjected to 100Ca + 0.05Ga following the NaOH treatment exhibited split peaks of approximately 347 and 350 eV in binding energy which were attributed to the Ca2p_3/2_ and Ca2p_1/2_ of CaO [37], and the peak range from 1115 to 1121 eV was deconvoluted into 1118.0, 1119.0 and 1116.5 eV of Ga2p_3/2_. It is reported in the literature that the peak around 1117–1118 eV is attributable to the Ga^3+^ of Ga_2_O_3_ [37,38], whereas the peak around 1119 eV is attributable to the Ga^3+^ or Ga^4+^ of the Ga–O located at the Ti^4+^ site in TiO_2_ [39]. The peak around 1116 eV was attributed to a metallic Ga bond [40]. Similar Ga 2p peaks were detected on the sample surfaces subjected to the 100Ga treatment after the NaOH treatment. These Ga peaks were not evidently changed by the subsequent heat and water treatments. The O 1s spectra on the same samples are shown in Figure 4. The spectra tailing to the higher binding energy side was deconvoluted to around 530 eV of Ti–O bond, 531 eV of physisorbed H_2_O, and 532 eV of basic Ti–OH [41]. Abundant Ti–OH peaks were observed on the sample surfaces subjected to 100Ca + 0.05Ga or 100Ga treatment following the NaOH treatment. A quantitative analysis revealed that the ratio of Ti–OH to Ti–O was 0.26 for the former and 0.24 for the latter. These values were severely reduced by the subsequent heat treatment, but recovered to some degree with the final water treatment.

**Figure 5:**
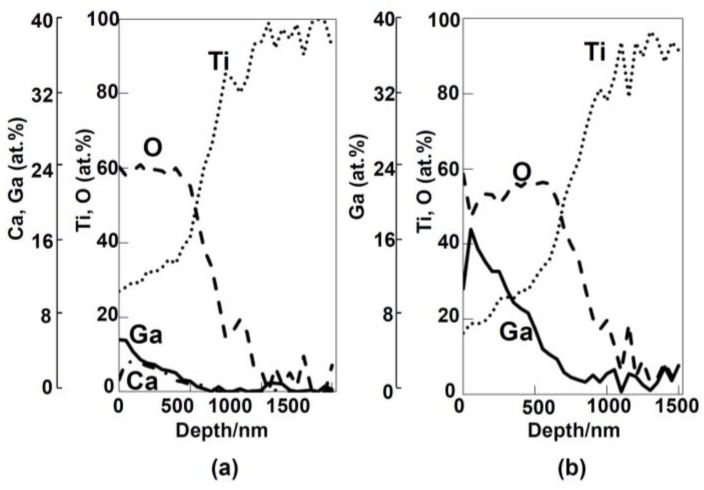
XPS depth profiles of Ti that was initially soaked in NaOH solution and then subjected to (**a**) 100Ca + 0.05Ga and (**b**) 100Ga treatment followed by heat and water treatments.

Figure 5 shows the depth profile of the XPS spectra of the Ti subjected to the 100Ca + 0.05Ga or 100Ga treatment after the NaOH treatment followed by heat and water treatments. Enrichment of Ca and Ga were observed near the surface in the former sample in addition to O, as shown in Figure 5a. They gradually decreased with increasing depth up to a thickness of approximately 1 μm. Substantially larger amounts of Ga were detected on the surface of the latter sample, as shown in Figure 5b.

### 2.2. Ion Release

**Figure 6:**
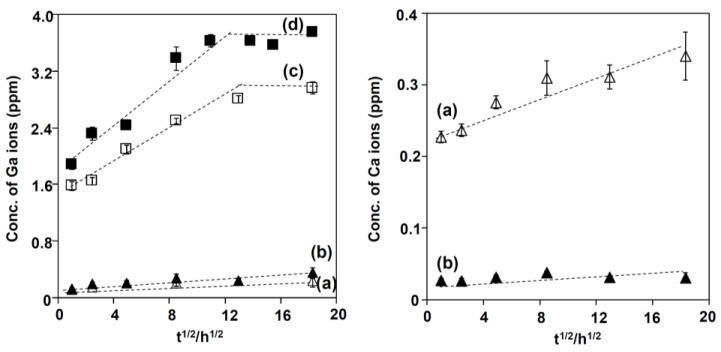
Concentrations of Ga and Ca ions measured by ICP (inductively coupled plasma emission spectroscopy), which was released from Ti subjected to (**a**) NaOH; 100Ca + 0.05Ga and heat treatment, and subsequent (**b**) water treatment; or (**c**) NaOH, 100Ga and heat treatment, and subsequent (**d**) water treatment as a function of square root of soaking time in PBS.

Figure 6 shows the concentration of the Ga and Ca ions released from the Ti subjected to 100Ca + 0.05Ga or 100Ga treatment after the NaOH treatment, followed by heat and water treatments, as a function of the square root of the soaking time in PBS. It can be seen in Figure 6c that the metal sample subjected to 100Ga and heat treatment following the NaOH treatment rapidly released 1.58 ppm of Ga ions within 1 h, and slowly released another 1.22 ppm up to 7 days in (a) proportion to the square root of the soaking time. Further release was not observed until 14 days. In total, 2.96 ppm of Ga ions was released. The Ga release increased up to 3.75 ppm when the metal was finally subjected to the water treatment, as shown in Figure 6d. In contrast, the metal subjected to 100Ca + 0.05Ga and heat treatment following the NaOH treatment released only a small amount of Ga ions, which was as low as 0.23 ppm, even after 14 days. The Ga release was slightly increased up to 0.35 ppm by the final water treatment, as shown in Figure 6b. A large amount of Ca ions, as high as 0.34 ppm, was released from the former sample, as seen in Figure 6a, but it decreased to 0.03 ppm with the final water treatment.

### 2.3. Apatite Formation

**Figure 7:**
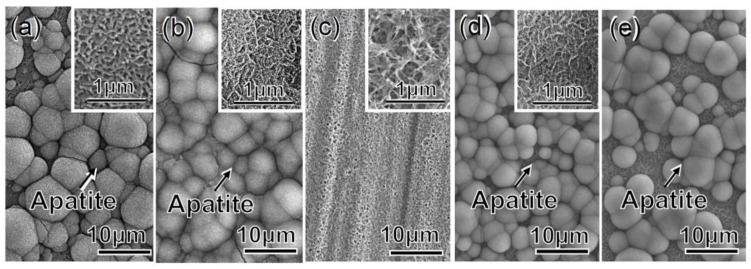
FE-SEM photographs of surfaces of Ti soaked in SBF for 3 days following (**a**) NaOH, 100Ca + 0.05Ga and heat treatments, and finally (**b**) water treatment, or (**c**) NaOH, 100Ga and heat treatments and finally (**d**) water treatment. Apatite formation of Ti subjected to the treatment of (**d**) followed by a storage under 95% relative humidity at 80 °C for 7 days is shown in (**e**). The insets in (**a**–**d**) show high-magnification images.

Figure 7 shows the FE-SEM photographs of the surfaces of the metal samples soaked in SBF after the chemical and heat treatments. When the metal was subjected to 100Ca + 0.05Ga and heat following the NaOH treatment, it formed many spherical precipitates on its surface within 3 days in SBF, as shown in Figure 7a. The amount of precipitate increased to fully cover the surface of the metal when additional water treatment was performed on the previously heat-treated samples, as shown in Figure 7b. Interestingly, when the metal was subjected to only 100Ga (without Ca) and heat following the NaOH treatment, no precipitates were observed on the metal surface. In contrast, considerable precipitate was observed on the surface of the heat-treated metal that was ultimately subjected to the water treatment, as shown in Figure 7d. The precipitate was also observed on the metal subjected to the same treatments followed by subsequent storage under 95% relative humidity at 80 °C for 7 days, as shown in Figure 7e. From high magnification images, it was quite clear that the precipitate that formed was composed of nano-sized crystals. No obvious difference was observed in terms of the crystal size of the apatite phase that formed on the various treated samples that were able to produce apatite in SBF.

**Figure 8:**
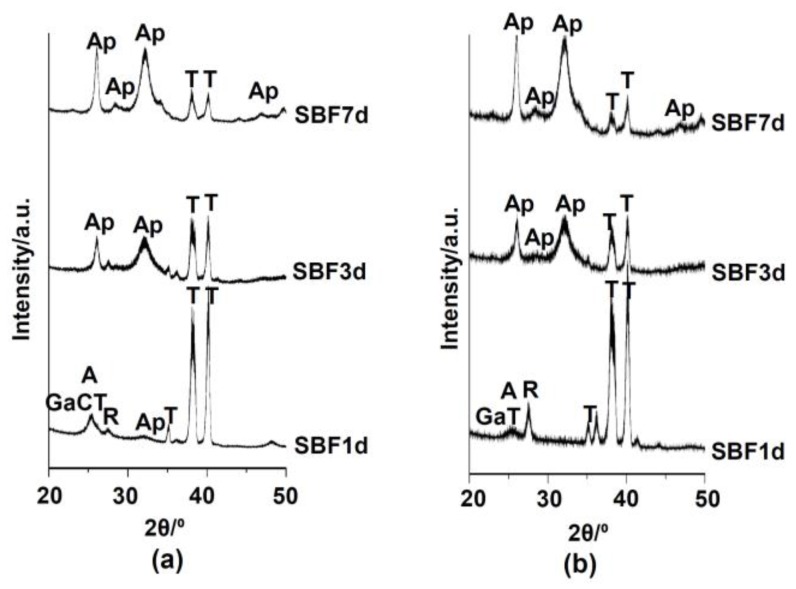
TF-XRD profiles of the Ti surfaces soaked in SBF for different days after (**a**) 100Ca + 0.05Ga, heat and water or (**b**) 100Ga, heat and water treatments following the NaOH treatment. T: α-Ti Ap: Apatite A: Anatase R: Rutile, Ga-CT: Ga*_x_*Ca_1−1.5*x*_Ti_4_O_9_, Ga*_x_*Ca_1−1.5*x*_Ti_2_O_4_, Ga*_x_*Ca_1−1.5*x*_Ti_4_O_5_, GT: Ga_2_TiO_5_.

Figure 8 shows the TF-XRD profiles of the samples that were soaked in SBF for various numbers of days after the ultimate water treatment. Broad peaks around 26° and 32° in 2θ appeared on the surface of the samples that were subjected to 100Ca + 0.05Ga or 100Ga treatment in a second solution within 1 or 3 days, respectively. This indicates that the spherical precipitate observed under SEM was composed of poorly crystalline apatite. The intensity of the apatite peaks increased with the increase of soaking time in SBF in both cases. No obvious changes were observed in the apatite that formed on the sample surfaces for the same soaking periods of 3 or 7 days, respectively. 

**Figure 9:**
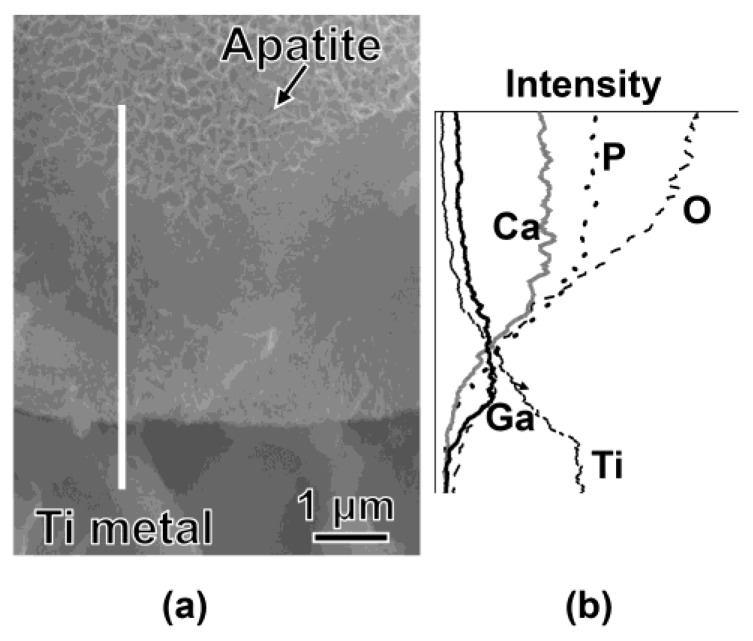
(**a**) FE-SEM photographs and (**b**) EDX line analysis of cross sections of Ti soaked in SBF for 7 days after 100Ga, heat and water treatments following the NaOH treatment. EDX line analysis was performed along with white line in the photographs.

**Figure 10:**
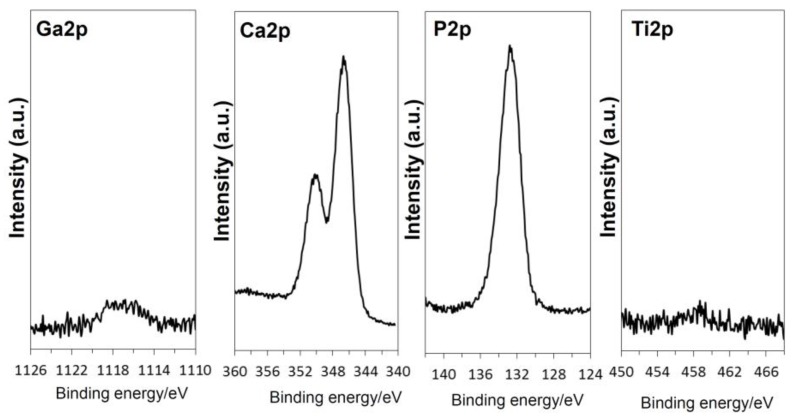
XPS profiles of Ga2p, Ca2p, P2p and Ti2p on Ti soaked in SBF for 7 days after NaOH-100Ga-heat-water treatment.

The sample formed with apatite by soaking in SBF for 7 days after the NaOH, 100Ga, heat and water treatments was subjected to cross-sectional FE-SEM observation and EDX line analysis to examine the Ga distribution on the surface of the metal (Figure 9). It was found from the FE-SEM photographs (Figure 9a) that apatite particles filled the spaces of the nano-structured surface layer and grew into a uniform layer approximately 4 μm in thickness. It was shown by EDX line analysis (Figure 9b) that a relatively large amount of Ga was detected in the region where Ti started to decrease, and it gradually decreased along with a decreasing Ti. A certain amount of Ga was detected even near the top surface, where Ca and P, which are components of apatite, were dominant. These results imply that Ga distribution was not limited in the surface GT layer, but also regions far from the GT layer. It was evident from the high-resolution XPS spectra of the Ga2p, Ca2p, P2p, and Ti2p taken on the top surface of the sample, shown in Figure 10, that a small amount of Ga was detected along with a large amount of Ca and P, even on the top surface of the apatite layer, while Ti was not detected. 

### 2.4. Antibacterial Activity

**Figure 11:**
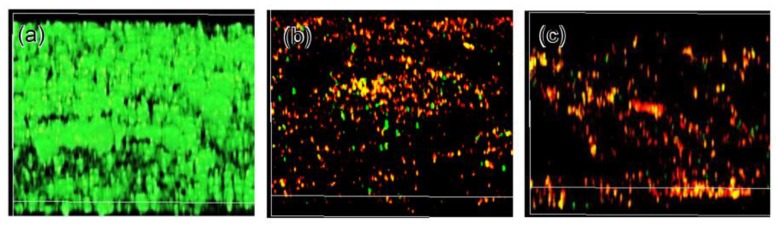
Confocal microscopy of Ti surfaces contacted with multi-resistant *Acinetobacter baumannii* (MRAB12) for 7 days (**a**) as-polished; (**b**) subjected to NaOH-100Ca + 0.05Ga-heat-water or (**c**) NaOH-100Ga-heat-water treatment. Green: live bacteria, Red: dead bacteria.

**Figure 12:**
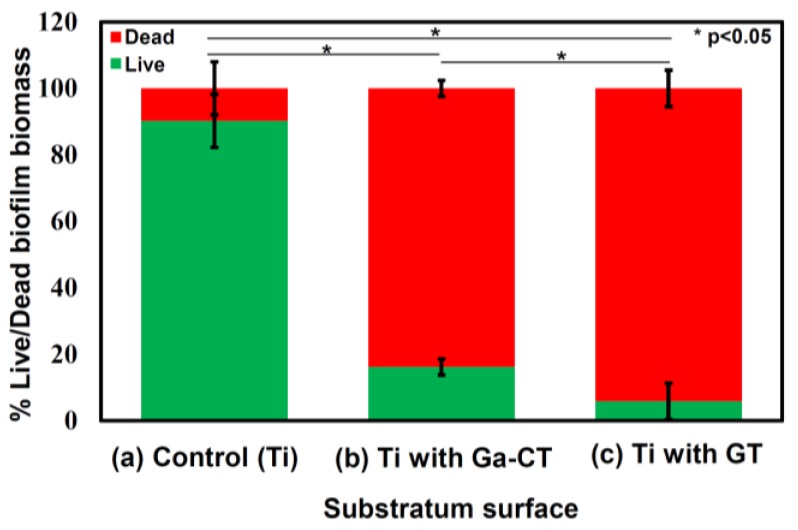
Live/Dead biofilm biomass on Ti surfaces contacted with multi-resistant *Acinetobacter baumannii* (MRAB12) for 7 days (**a**) as-polished; (**b**) subjected to NaOH-100Ca + 0.05Ga-heat-water or (**c**) NaOH-100Ga-heat-water treatment.

Figure 11 shows the confocal microscopy images of the Ti surfaces containing multi-resistant *Acinetobacter baumannii* (MRAB12). It was evident that the bacteria proliferated to form dense film on the surface of as-polished Ti, while they were almost entirely killed on the surfaces of Ti with Ga–CT or GT. Quantitative analysis revealed that the percentage of Live biofilm biomass on the control sample was significantly higher (*p* < 0.05) at 87.7% ± 4% in comparison to both the samples with Ga–CT (16.2% ± 5.5%) and those with GT (5.8% ± 2.9%), as shown in Figure 12. Concurrently, the percentage of Dead biofilm biomass was significantly higher (*p* < 0.05) on both the samples with Ga–CT (83.8% ± 5.5%) and those with GT (94.2% ± 2.4%). 

## 3. Discussion

The initial NaOH treatment formed a fine network layer that consisted of nano-sized sodium hydrogen titanate, Na*_x_*H_2−*x*_Ti_3_O_7_, on the surface of the metal. Since sodium hydrogen titanate (SHT) assumes a layered structure [34], its Na ions were easily substituted by Ca and Ga ions so as to form a gallium-containing calcium hydrogen titanate (Ga–CHT) when soaked in a mixed solution of CaCl_2_ and GaCl_3_ solution. It is evident from the XPS profiles of Ca2p and Ga2p in Figure 3 that all of the Ca ions and most of the Ga ions were located in interspaces of Ga–CHT, whereas some Ga ions were located at the Ti^4+^ site. When the Ga–CHT with a Ga/Ca ratio of 0.26 was subjected to the heat treatment, it was transformed into gallium-containing calcium titanate (Ga–CT), rutile and anatase, as shown in Figure 2d. Interestingly, the metal treated thusly formed apatite on its surface in SBF within 3 days, as shown in Figure 7a, even without the final water treatment reported to be essential to induce apatite formation on Ti formed with calcium titanate (CT), Sr-containing calcium titanate (Sr–CT) and Mg-containing calcium titanate (Mg–CT) [17,18,34]. The apatite formation on Ti with Ga–CT is probably due to the relatively large capacity for Ca ion release, as shown in Figure 6a, compared to that of Ti with CT, Sr–CT, and Mg–CT. The large capacity of Ca ion release on Ga–CT might be due to the Ga ions located at the Ti^4+^ site of Ga–CT, resulting in a distortion in the tight CT structure. Thus, most of the Ca ions were replaced with H_3_O^+^ ions when the treated metal was subsequently subjected to the final water treatment, as shown in Table 1. As a result, Ca ion release was significantly decreased, as shown in Figure 6b, which is in contrast to the slight decrease in Ca content and increase in Ca ion release on the Ti formed with CT [18]. Nevertheless, the apatite formation of Ga–CT was further increased by the final water treatment, as shown in Figure 7b. Thus, the increased apatite formation was not attributed to Ca ion release. This was also not attributed to the crystalline phases that were not obviously changed by the final water treatment, as shown in Figure 2d,e, but rather is probably due to the abundant Ti–OH groups that were preferable for apatite nucleation [42], as shown in Figure 4c. It was clear, however, as shown in Figure 6a,b, that the capacity of Ga ion release on Ga–CT was poor, even after the final water treatment.

When the Ti was soaked in 100 mM GaCl_3_ solution after the NaOH treatment, 8.0% Ga was incorporated into the metal surface, forming gallium hydrogen titanate (GHT). Most of the Ga ions were located in the interspaces of GHT whereas some were at the Ti^4+^ site, as seen in the case of Ga–CHT. The GHT became dehydrated and transformed into gallium titanate (GT), rutile and a small amount of anatase by the heat treatment. The treated metal did not form apatite in SBF within 3 days, but formed a marked amount apatite particles after the final water treatment, as shown in Figure 7c,d. The increased apatite formation by the final water treatment might be attributable to the increased Ga ion release as well as abundant Ti–OH groups, as shown in Figure 4c and Figure 6d. The concentration of Ga in PBS become 1.88 ppm after 1 h, and slowly increased up to 3.75 ppm after 7 days, which remained there until after 14 days. When the treated metal was soaked in SBF, it is assumed that Ga ions were also released into SBF so as to increase the pH of the surrounding fluid via an exchange with H_3_O^+^ ions, and therefore formed Ti–OH groups on the metal surface, as in the case of the Na ions in the sodium titanate that was formed on Ti [43]. The Ti–OH groups become negatively charged in an alkaline environment [44], and combine with the positively charged Ca ions and, subsequently, negatively charged phosphate ions in the SBF. As a result, an amorphous calcium phosphate is formed, which is eventually transformed into crystalline, bone-like apatite [45]. Thus, the formed apatite becomes integrated with the nanostructured surface layer by growing into a uniform layer of about 4 μm in thickness, as shown in Figure 9. It was evident from the EDX line analysis in Figure 9 and XPS profiles on apatite far from the surface layer in Figure 10 that a small amount of Ga ions was taken into the apatite precipitated in SBF. It is reported that Ga ions in physiological saline solution supersaturated with Ca and PO_4_ preferentially adsorbed on apatite crystal to reduce the rates of direct precipitation of apatite, transformation of amorphous calcium phosphate to apatite and growth of apatite seed crystals when the concentrations of Ga ions in the solution were more than 7, 17.5, and 1.75 ppm, respectively [46]. In the present study, no distinguishable differences were observed in terms of the crystallinity of the apatite formed on Ti with Ga–CT and GT regardless of the difference in their capacities for Ga release, as can be seen from Figure 7 and Figure 8. This might be due to the relatively small Ga ion amounts (maximum at 3.75 ppm) compared to the reported value. The capacity of apatite formation on the metal, treated in this manner, was essentially maintained, even in 95% relative humidity at 80 °C for at least 1 week, as shown in Figure 7e.

It has been shown that a rod comprised of the NaOH- and heat-treated Ti was able to be pulled out of its site only by being accompanied with a bone fragment when it was implanted into an intramedullary canal of rabbit femur for 12 weeks [47]. A large number of bone fragments remained on the CT layer that formed on Ti by Ca-heat treatment when the metal was detached from the rabbit tibia after an implantation period of 4 to 26 weeks [48]. This indicates that the bonding strength between the substrate and the surface layer formed by the NaOH-heat or Ca-heat treatment was higher than the tensile strength of the bone. The strong bonding to bone was attributed to the capacity of apatite formation of the treated metals in both cases [47,48]. Thus, it is expected that Ti with Ga-CT or GT formed by the present treatments will also be strongly bonded to bone due to its capacity for apatite formation.

It was recently shown that the Ga-doped Ti produced by anodic spark deposition, releasing about 0.2 ppm of Ga ions into PBS, achieved better bacterial inhibition against one strain (DSM 30007) and two clinical isolates (AB1 and AB2) of *Acinetobacter baumannii* than Ag-doped Ti by the same method [24]. The antibacterial mechanism of Ga was suggested to be the “Trojan horse” mechanism in which Ga^3+^ ions effectively compete with Fe^3+^ ions for binding to siderophores, thus interrupting crucial Fe-dependent metabolic pathways in bacteria due to the biological similarity of Ga^3+^ to Fe^3+^ ions [24]. In the present study, Ti with an increased capacity for Ga ion release was prepared by the simple chemical and heat treatments. The Ti treated with Ga–CT or GT exhibited strong bacterial inhibition of *Acinetobacter baumannii* clinical isolates (MRAB12), as shown in Figure 11 and Figure 12. The inhibitory effect was much higher for the latter, which released a greater amount of Ga. In addition, the effect of Ga ions on bone resorption and osteoclasts has been reported by many researchers. Hall et al. reported that 0.027 to 27 ppm of Ga ions derived from gallium nitrate produced a concentration-dependent inhibition of bone resorption by the osteoclasts isolated from neonatal rat long bones and cultured on slices of rat cortical bone [49] Verron et al. reported that 0.7 to 7 ppm of Ga ions derived from gallium nitrate inhibited the in vitro resorption activity of RBC and induced a significant decrease in the expression level of transcripts coding for osteoclastic markers in RAW 264.7 cells [27]. Studies have shown that gallium is adsorbed onto the surface of bone, where it is effective in blocking osteoclastic resorption. At antiresorptive concentrations, gallium does not appear to be cytotoxic to osteoclasts [28], or to act as a cellular metabolic inhibitor [29]. It is expected that the Ti that formed the Ga–CT or GT layer as the result of the NaOH-100Ca + 0.05Ga-heat-water or NaOH-100Ga-heat-water treatment in the present study would exhibit inhibitory effect on bone resorption and microbial activity so as to increase bone density and prevent infection in the living body, since it releases a concentration of Ga ions comparable to or greater than the reported values [27,49].

## 4. Materials and Methods 

### 4.1. Surface Treatment

Pure Ti (Ti > 99.5%; medical grade, ISO5832-2, Nilaco Co., Tokyo, Japan) with dimensions of 10 × 10 × 1 mm^3^ was abraded with #400 diamond plates, washed with acetone, 2-propanol and ultrapure water in an ultrasonic cleaner for a period of 30 min, then dried at 40 °C. The Ti samples were soaked in 5 mL of 5 M NaOH (Reagent grade; Kanto Chemical Co., Inc., Tokyo, Japan) aqueous solution at 60 °C in an oil bath, then shaken at a speed of 120 strokes/min for a period of 24 h followed by gentle rinse with ultrapure water for a period of 30 s. They were subsequently soaked in 10 mL of a mixed solution of 100 mM CaCl_2_ (Reagent grade; Kanto Chemical Co., Inc., Tokyo, Japan) and *X* mM GaCl_3_ (Reagent grade; Kanto Chemical Co., Inc., Tokyo, Japan) at 40 °C, where *X* is a range from 0.01 to 0.1 and designated as “100Ca + *X*Ga”, shaken at a speed of 120 strokes/min for a period of 24 h, washed and dried in a similar manner. One metal soaked in 10 mL of 100 mM GaCl_3_ after the NaOH treatment was also prepared and designated as “100Ga”. They were subsequently heated to 600 °C at a rate of 5 °C/min and maintained at 600 °C for 1 h, then allowed to be cooled in a Fe–Cr electrical furnace. After the heat treatment, they were soaked in 10 mL of hot water at 80 °C, shaken at a speed of 120 strokes/min for a period of 24 h, washed and dried. 

### 4.2. Surface Analysis

#### 4.2.1. Scanning Electron Microscopy

The chemical- and heat-treated samples were coated with a Pt–Pd thin film, then their surfaces and cross sections were observed under field emission scanning electron microscopy (FE-SEM: S-4300, Hitachi Co., Tokyo, Japan) with a voltage of 15 kV.

#### 4.2.2. Energy Dispersive X-ray Analysis

The surface chemical composition of the samples was analysed with an energy dispersive X-ray spectrometer (EDX: EMAX-7000, Horiba Ltd., Kyoto, Japan) by using 9 kV–K for the Ca, Ga, O and Ti in five areas. Their averaged value was used for analysis.

#### 4.2.3. Thin-Film X-ray Diffraction and Fourier Transform Confocal Laser Raman Spectrometry

The surfaces of the samples after the chemical and heat treatments were analysed by a thin-film X-ray diffractometer (TF-XRD: model RNT-2500, Rigaku Co., Tokyo, Japan) and Fourier transform confocal laser Raman spectrometer (FT-Raman: LabRAM HR800, Horiba Jobin Yvon, Longjumeau, France). In TF-XRD analysis, a CuKα X-ray source was used and the incident beam was fixed to an angle of 1° against the sample surface. The measurement was performed at 50 kV and 200 mA. In the FT-Raman measurements, an Ar laser with a wavelength of 514.5 nm was selected as the laser source and its laser power excitation was maintained to be 16 mW.

#### 4.2.4. X-ray Photoelectron Spectroscopy

The distribution of elements with a near-surface depth in the metal samples that has been subjected to the chemical and heat treatments was analyzed using X-ray photoelectron spectroscopy (XPS, PHI 5000 Versaprobe II, ULVAC-PHI, Inc., Kanagawa, Japan) under Ar sputtering at a rate of 4 nm per min (SiO_2_ conversion). An Al-Kα radiation line was used as the X-ray source with the XPS take-off angle at 45 degrees so that the system is able to detect photoelectrons to a depth of 1 to 5 nm from the surface. The calibration of the measured spectra was performed by reference to the C1s peak of the surfactant CH_2_ groups on the substrate occurring at 284.8 eV in binding energy.

### 4.3. Ion Release

The samples after the chemical and heat treatments were soaked in 2 mL of phosphate-buffered saline (PBS) at a specific concentration (Na^+^ 158.14, K^+^ 1.06, Cl^−^ 155.17, and HPO_4_^2−^ 4.03 mM) the pH adjusted to 7.4 at 36.5 °C. They were gently shaken at a speed of 50 strokes/min for predetermined periods of up to 7 days. After being removed from the PBS, the Ca and Ga ion concentrations in the PBS were measured by inductively coupled plasma emission spectroscopy (ICP, SPS3100, Seiko Instruments Inc., Chiba, Japan). Three samples were independently prepared for each soaking condition, and the values of average and standard deviation were calculated.

### 4.4. Soaking in SBF

Apatite formation of the samples after the chemical and heat treatments were examined by soaking in 24 mL of SBF that has ion concentrations (Na^+^ 142.0, K^+^ 5.0, Ca^2+^ 2.5, Mg^2+^ 1.5, Cl^−^ 147.8, HCO_3_^−^ 4.2, HPO_4_^2−^ 1.0, and SO_4_^2−^ 0.5 mM) nearly equal to those of human blood plasma at 36.5 °C for various periods of up to 7 days. The SBF was prepared by dissolving reagent grade NaCl, NaHCO_3_, KCl, K_2_HPO_4_·3H_2_O, MgCl_2_·6H_2_O, CaCl_2_, and Na_2_SO_4_ (Nacalai Tesque Inc., Kyoto, Japan) in ultrapure water, and then buffered at pH = 7.4 with tris(hydroxymethyl)aminomethane (CH_2_OH)_3_CNH_2_ and 1 M HCl (Nacalai Tesque Inc., Kyoto, Japan) at 36.5 °C [50]. After being removed from the SBF, the samples were gently rinsed with ultrapure water and dried, then their surface apatite formation was analysed using TF-XRD, FE-SEM and EDX. 

### 4.5. Antibacterial Activity Test

#### 4.5.1. Bacterial Culture

Clinically isolated strains of Multidrug Resistant, *Acinetobacter baumannii* (MRAB12, isolated from patients’ catheters, Concord Hospital, Sydney, Australia). The bacteria were grown in Tryptone Soy Broth (TSB) over a period of 16 h at 37 °C and 150 rpm. Following the growth period, a 1:10 dilution of bacterial suspension was made in TSB for inoculation of the surfaces.

#### 4.5.2. Biofilm Formation

Ti samples as-polished or treated in the manner described in Section 2.1 were placed in 24-well cell culture plates. One millilitre of the diluted bacterial suspension was used to submerge the samples so as to allow adequate contact of the bacterial suspension with the surfaces. Biofilm was allowed to form over a period of 7 days, with media being replaced with 1 mL of fresh media on Day 3 and 5 to ensure sufficient nutrient availability for bacterial growth. On Day 7, media was removed and the samples rinsed with 1 × phosphate buffer saline (PBS) thrice, in order to remove any planktonic or loosely attached bacteria from the surface. Biofilms were then stained using the fluorescent nucleic acid stain SYTO-9 for detection of live cells, and with the cell-impairment nucleic acid stain Propidium iodide (PI) for detection of the dead cells (i.e., cells with compromised membranes) (the LIVE/DEAD Bac-Light Bacterial Viability Kit, Molecular Probes, Thermo Fisher Scientific, Waltham, Massachusetts, USA ). The stain mixture was prepared as per the manufacturer’s instructions and added directly to the surfaces, then allowed to incubate under dark at room temperature for a period of 30 min.

#### 4.5.3. Confocal Microscopy and Analysis

Live/dead stained biofilm samples were imaged using a Nikon C2 Confocal microscope (Nikon Corp., Tokyo, Japan) with laser settings of 473 nm and 559 nm for green (live) and red (dead) staining, respectively. Fiji ImageJ software (National Institutes of Health, USA) was employed to generate images and quantify the biovolume as well as the percentage of live and dead biofilm.

#### 4.5.4. Statistical Analysis

Statistical analysis was performed using GraphPad Prism 7.02 software (GraphPad Software, Inc., La Jolla, CA, USA). The differences in the means between the sample groups were analysed using Tukey’s multiple comparisons test, with a *p*-value < 0.05 indicating a statistically significant difference. 

## 5. Conclusions

We have developed a simple and cost-effective technology to fabricate next-generation interfaces for titanium implants that:

- Notably enhance mineralisation of the matrix directly on the surface, thus supporting bone tissue formation.
- Sustainably release gallium ions, which have positive effects on bone formation and bone quality.
- Strikingly reduce biofilm formation on the surface.Figur

The developed technology is significant, because there are no efficacious treatment options available for patients living with infected implants, hence there is a huge unmet need. Furthermore, the proposed bioactive and antimicrobial surfaces represent a rare breakthrough towards the development of true multifunctional surfaces. We have also demonstrated that the release of the gallium can be tuned to adjust the strength of antimicrobial activity to individual patients/needs and it allows us to use our two-prong approach to eliminate infections and to promote tissue regeneration. This concept is fully translatable for other devices that suffer from high rate of infections and limited integration within bodily environment.

Specifically, our interfaces are composed of Ga-containing calcium titanate (Ga–CT) or gallium titanate (GT) accompanied by rutile and anatase. Ga–CT samples encouraged the apatite formation in SBF within 3 days, but released Ga ions at a low level: 0.23 ppm in PBS within 14 days. In contrast, GT interfaces released slowly 3.75 ppm of Ga ions up to 7 days and formed apatite within 3 days after the final water treatment. Although, a trace amount of Ga was incorporated into the apatite formed on GT samples, it had no effect on the apatite crystal structure. The inhibitory effect on micro and bioactivity was shown got both Ga–CT and GT, with some increase of ABC observed for GT samples. It can be expected that multifunctional interfaces will be particularly useful for orthopaedic and dental implants since it will facilitate direct disposition of the bone at the surface, thus it will promote desired implant integration, while the sustained release of Ga ions will prevent infections and inhibit bone resorption. 

## Figures and Tables

**Table 1:** Chemical compositions of the surface layers of Ti metal subjected to NaOH, Ca + Ga, heat and water treatments, which were analysed by EDX.

Treatment	Element (at. %)					Ga/Ca Ratio
	O	Ti	Na	Ca	Ga	
Untreated	3.1	96.9	0	0	0	-
NaOH	66.8	27.7	5.5	0	0	-
NaOH-100Ca + 0.01Ga	68.6	27.6	0	3.5	0.3	0.09
NaOH-100Ca + 0.05Ga	67.9	29.2	0	2.3	0.6	0.26
NaOH-100Ca + 0.10Ga	68.3	27.7	0	1.2	2.8	2.33
NaOH-100Ga	69.3	22.8	0	0	8.0	-
NaOH-100Ca + 0.05Ga-heat	68.5	28.7	0	2.3	0.6	0.26
NaOH-100Ca + 0.05Ga-heat-water	68.6	29.5	0	1.2	0.7	0.58
NaOH-100Ga-heat	68.4	23.5	0	0	8.1	-
NaOH-100Ga-heat-water	69.8	22.4	0	0	7.8	-
